# Modified Percutaneous Achilles Tendon Lengthening by Triple Hemisection for Achilles Tendon Contracture

**DOI:** 10.1155/2019/1491796

**Published:** 2019-11-07

**Authors:** Yangjing Lin, Jin Cao, Changgui Zhang, Liu Yang, Xiaojun Duan

**Affiliations:** Center for Joint Surgery, Southwest Hospital, Third Military Medical University (Army Medical University), Chongqing 400038, China

## Abstract

**Background:**

Both percutaneous Achilles tendon lengthening by triple hemisection and the traditional open Z-lengthening are effective methods for Achilles tendon contracture. This study aims to evaluate the efficacy and safety of this new therapeutic method, which is based on the percutaneous sliding technique with three hemi-cuts in the tendon, as compared with the traditional open Z-lengthening.

**Methods:**

Retrospective analysis of the Achilles tendon contracture cases in our hospital between January 2010 and September 2016 was conducted. Twenty-five cases received percutaneous Achilles tendon lengthening (group A), and 30 patients who underwent open Z-lengthening during the same period were in the control group (group B). Operative time and hospital stay were statistically analyzed. Incision complication, equinus recurrence rate and Achilles tendon rupture morbidity were recorded. The function was assessed by American Orthopaedic Foot & Ankle Society (AOFAS) score. All cases in group A received Magnetic Resonance Imaging (MRI) of ankle preoperatively and in the follow-ups.

**Results:**

The mean follow-up period was 42.04 months in group A and 61.7 months in group B. The entire operative time and the mean hospitalization days were lower in group A than in group B. No incision and infection complication occurred in group A. The infection rate in group B was 3.3%. Equinus recurrence rate was 4% in group A and the equinus recurrence rate in group B was 21.4%. In group A, the mean AOFAS score increased from 64 ± 10.16 points preoperatively to 96.08 ± 3.17 at final follow-up, while the score in group B increased from 63.48 ± 6.2 points to 85.4 ± 10.3. MRI showed continuity of the Achilles tendon and homogeneous signal in group A.

**Conclusion:**

Modified surgery can significantly reduce the risk of Achilles tendon rupture, provide better balance in soft tissue strength between ankle dorsiflexion and ankle plantarflexion, helping to avoid recurrence of the deformity.

## 1. Introduction

Achilles tendon contracture, which affects the dorsiflexion of ankle, is common in clinic. Many factors including neurological abnormalities, trauma and developmental abnormalities contribute to the risk of Achilles tendon contracture [1]. Achilles tendon lengthening is an effective method for treating Achilles tendon contracture. The goal of surgery is to improve the dorsiflexion of ankle and correct deformity [2]. The traditional open Z-lengthening has an advantage in getting enough lengthening; however, incision complication could be frequently observed, some even result in other serious complications such as adhesion, pain, scarring, or infection [3]. Percutaneous Achilles tendon lengthening by triple hemisection was first employed by Hoke (1931) [4]. The advantages of this method are a shorter hospital stay, reducing the incidence of incision complication and negligible scarring [5]. Percutaneous Achilles tendon lengthening is now globally accepted as an easy and efficient treatment for Achilles tendon contracture [6]. However, the Achilles tendon rupture caused by percutaneous Achilles tendon lengthening is gradually paid attention to, especially if the Achilles tendon contracture is very serious. So it's worth going for a further discussion whether the open Z-lengthening can be entirely displaced by the percutaneous Achilles tendon lengthening.

Our study aims to provide a new therapeutic theory which bases on the percutaneous sliding technique with three hemi-cuts in the tendon. Although there are still three small incisions, our fundamentals are different from Hoke's theory [4]. In this study, Achilles tendon parenchymal at distal level was hemisected through the most distal incision, Achilles tendon parenchymal at proximal level was hemisected through the middle incision, then Achilles tendon lengthening was accomplished by the sliding tendon under forceful dorsiflexion. Besides, part of the reason for Achilles tendon contracture is soft tissue imbalance which the ankle strength in plantarflexion is stronger than in dorsiflexion. It can better achieve soft tissue balance and reduce the recurrence of Achilles tendon contracture when Achilles tendon strength in plantarflexion is weakened. Thus in this study, the partial aponeurosis of gastrocnemius was cut off at most proximal incision in order to weaken Achilles tendon strength in plantarflexion.

In this study, we performed a retrospective analysis of the Achilles tendon lengthening cases in our hospital in recent years and evaluated the efficacy and safety of the new percutaneous Achilles tendon lengthening, as compared with the traditional open Z-lengthening, hoping to provide reference for further promotion of this technology.

## 2. Material and Methods

The inclusive criteria were, (1) patients older than 3 years; (2) severe Achilles tendon contracture and equinus; (3) the history was more than 6 months; (4) initial Achilles tendon lengthening. The exclusion criteria were listed below. (1) Patients younger than 3 years. (2) The course of Achilles tendon contracture was within 6 months. (3) Patient had suffered from the Achilles tendon lengthening. (4) Obvious contracture of other soft tissues occurred in combination, such as compartment syndrome or extensive scar tissues in calf. (5) Operation was needed to perform jointly by osteotomy or tendon transposition, such as flat foot. (6) Ankle joint was damaged obviously, such as Charcot's joint or Hemophilic arthritis. (7) Skin soft-tissue infection occurred in the lower limb. (8) Coagulation abnormalities.

The Achilles tendon contracture cases in our hospital between January 2010 and January 2016 was conducted. 25 cases were in percutaneous Achilles tendon lengthening group (group A), and 30 patients who underwent open Z-lengthening during the same period were in the control group (group B) (Table 1).

All operations in group A were performed by one senior surgeon and cases in group B were performed by the other senior surgeons. Achilles tendon contracture was judged by Silfverskiold test [7], rather than gastrocnemius contracture. Routine ankle radiographs were conducted to rule out the unusual bone structure. Degeneration and structural abnormalities in Achilles tendon should be known by Magnetic Resonance Imaging (MRI).

### 2.1. Surgical Technique

For percutaneous Achilles tendon lengthening, anesthesia of lumbar plexus-sciatic nerve block and thigh tourniquet were used. The patient was placed at supine position, with feet a little away from the edge of the table and dorsiflexion movement of ankle could be easily achieved. After skin preparation and draping, the boundary of Achilles tendon and incision position was marked. Medial Achilles tendon strength needed to be weakened if the ankle was varus deformity, so the most distal incision was placed at the medial site 0.5 cm away from the proximal calcaneus. The middle incision was placed at the lateral site 5 cm away from the most distal incision. The distance between the most distal incision and the middle incision could expand to 8–10 cm based on the degree of Achilles tendon contracture (the site was away from soleus). The most proximal incision was on the surface of the gastrocnemius aponeurosis. The partial aponeurosis of gastrocnemius was cut off at most proximal incision in order to weaken Achilles tendon strength in plantarflexion. The distance between the most proximal incision and middle incision was not constant. Three 0.5-cm longitudinal incisions were made, then subcutaneous dissection was performed by hemostatic forceps. A No. 15 blade was inserted longitudinally in the middle tendon at three levels. Next, blunt dissection was carried out during the course of operation; the blade was rotated 90 degrees and the Achilles tendon was transected over half of the tendon. If the ankle was valgus deformity, the orientation of the triple incisions would be in the opposite direction. The hemisections were performed with knee in extension, then the dorsiflexion strength of ankle was gradually increased, and Achilles tendon lengthening was accomplished by the sliding tendon. When the degree of ankle joint dorsiflexion increased more than 100°, the purpose of surgical treatment was achieved.

**Figure 1:**
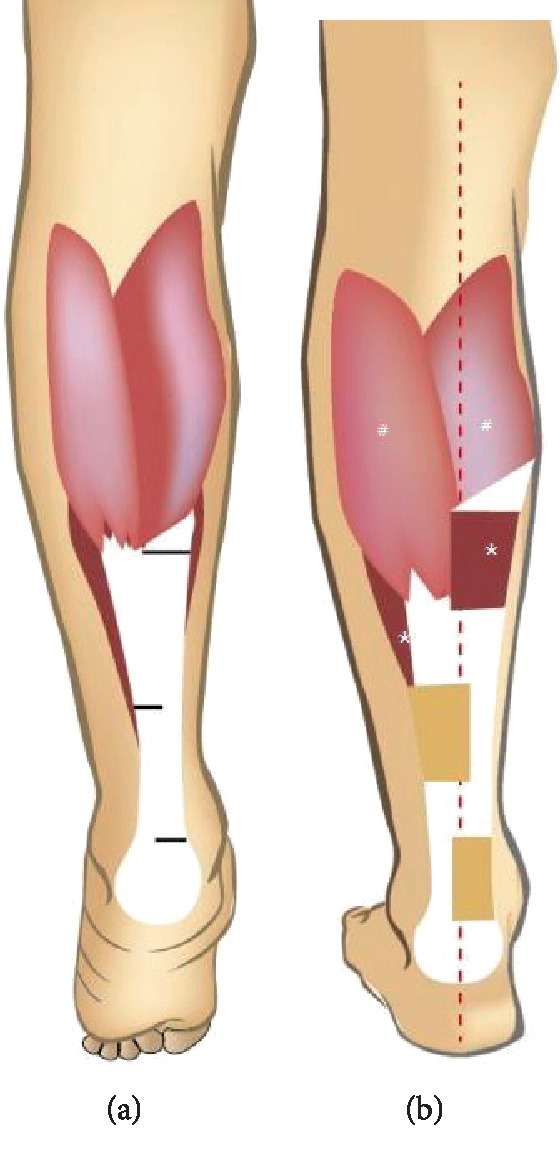
Pattern diagram of the operation. The three incisions (black line) were placed at Achilles tendon (a) and Achilles tendon lengthening was accomplished by the sliding tendon (b). Gastrocnemius (#). soleus (∗).

**Figure 2:**
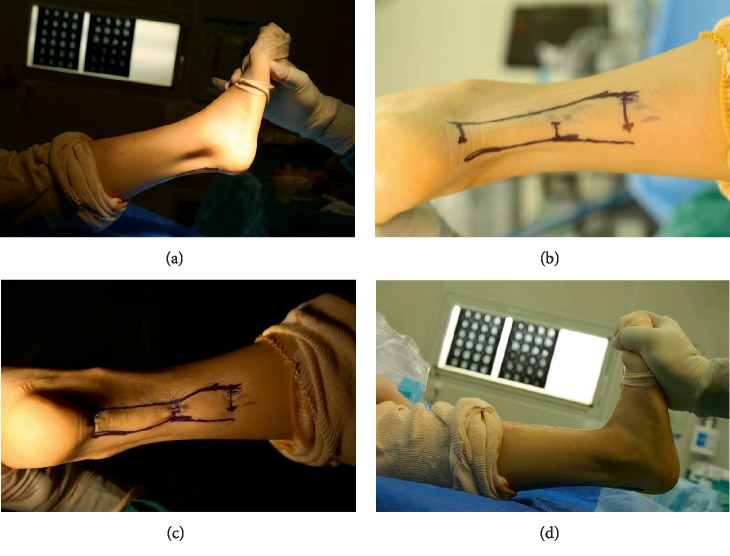
The main procedures of the surgery. Achilles tendon contracture preoperatively (a). The three incisions and the outline of the tendon were marked on the skin before the operation (b). Wound was closed after operation (c). The degree of ankle joint dorsiflexion increased more than 100° postoperatively (d).

If the Achilles tendon was not lengthened, the cut range should be increased when resection range was probed with hemostatic forceps (Figures 1 and 2).

For the traditional open Achilles tendon lengthening, anesthesia of lumbar plexus-sciatic nerve block and thigh tourniquet were used. The patient was placed at prone position, and feet were a little away from the edge of the table. After skin preparation and draping, a posteromedial incision was made. The Achilles tendon was exposed and cut by Z type, then the Achilles tendon was sutured at most dorsiflexion position of ankle [8].

In the last step, the wound was closed and the ankle was kept in appropriate position using a below-knee cast in both groups.

### 2.2. Postoperative Treatment

The ankle was kept in appropriate position for 4 weeks with the below-knee cast. Then partial-weight-bearing crutch was allowed with ankle-foot boots for 6 weeks. Patients were encouraged to perform ankle flexion and extension exercises in painless conditions. At 10 weeks postoperatively, patients were allowed to gradually participate in swimming and riding.

### 2.3. Assessment of Results

Operative time and hospital stay were statistically analyzed. Incision complication, equinus recurrence rate and Achilles tendon rupture morbidity were recorded. The function of ankle was assessed by AOFAS scores. All cases in group A received preoperative MRI and some patients received MRI examination in follow-up study. The quantitative data was described by using mean, and paired-sample t-tests was used (version 18.0; IBM, Chicago, IL, USA). Chisquare test was used to statistically analyze the incidence rate. Significance level was set as *P*< 0.05.

## 3. Results

In this study, all 25 patients in group A and 28 in group B were followed. The mean follow-up period was 42.04 months (range 12–101 months) in group A and 61.7 months (range 12–103 months) in group B. There was no significant difference between two groups in age, sex, deformity and the course of the disease.

**Figure 3:**
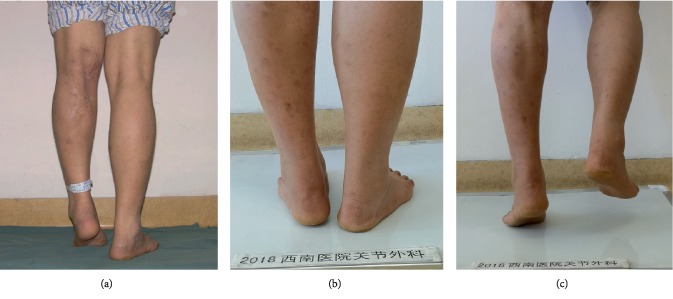
One typical case in group A. Male, 35-year-old. Right equinus was showed when standing preoperatively (a). After percutaneous Achilles tendon lengthening, right equinus disappeared when standing at 88 months postoperatively (b). The patient can perform single-limb heel rise with the lower extremity at 88 months postoperatively (c).

**Figure 4:**
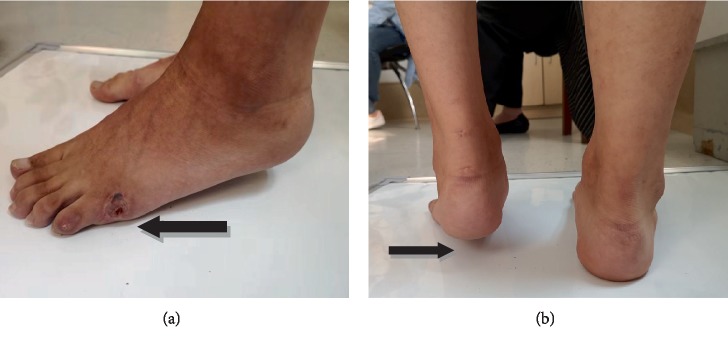
The only recurrence case in group A. Male, 24-year-old, suffered from equinus recurrence one year after percutaneous Achilles tendon lengthening because of the infection at his fifth metatarsal (a, arrowed site), and then his left foot was not on the floor (b, arrowed site).

The mean operative time was 10.8 ± 5.02 minutes in group A and 35.21 ± 13.58 minutes in group B. The mean hospitalization days was 3.08 ± 1.35 days in group A and 6.46 ± 2.34 days in group B. Both the above data in group A were lower than those in group B (*P* < 0.05). In group A, the mean AOFAS score increased from 64 ± 10.16 preoperatively to 96.08 ± 3.17 at final follow-up (Figure 3), while the AOFAS score in group B increased from 63.48 ± 6.2 to 85.4 ± 10.3. Equinus recurrence rate in group A was 4% (one patient) and only 8.3% of the patients (two patients) showed foot drop when they walked up stairs. The patient in the group A suffered from equinus recurrence one year after surgery because of the infection at his fifth metatarsal, and then his feet were not on the floor for nearly 6 months (Figure 4). Equinus recurrence rate in group B was 21.4% and 32.1% of the patients (9 patients) showed foot drop when they walked up stairs. Equinus recurrence rate between A group and B group was significant (*P* < 0.05).

No incision and infection complication occurred in group A. The infection rate in group B was 3.3%. There was no blood vessel, nerve injury and Achilles tendon rupture in both groups.

**Figure 5:**
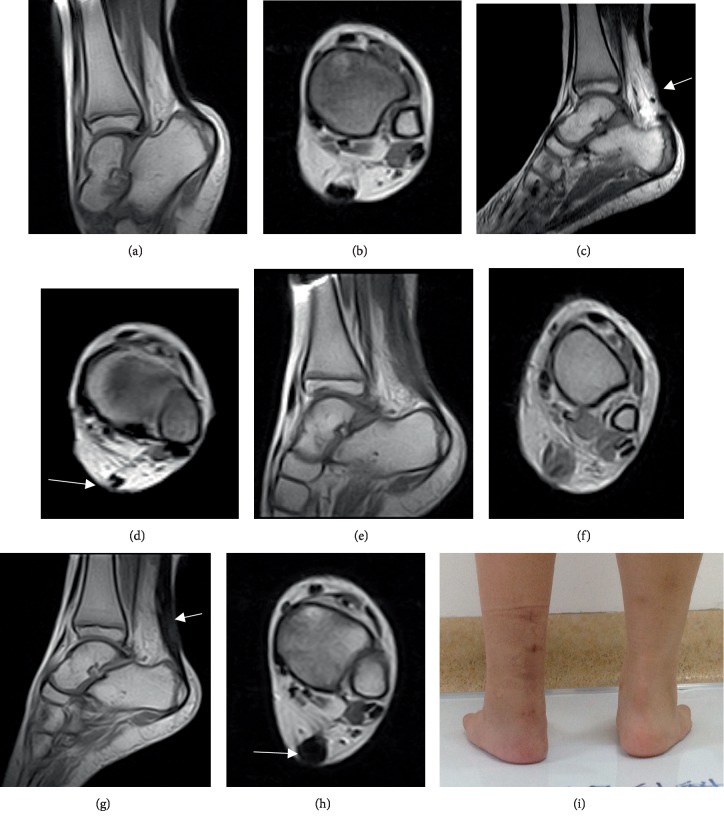
MRI images and photo of a 8-year-old female. Preoperative MRI showed Achilles tendon and equinus (a, b). MRI showed Achilles tendon was transected over half of the tendon while tendon was not totally torn (arrowed site) after 2 days of percutaneous Achilles tendon lengthening (c, d). Wounds in tendon were healed at 2 months postoperatively (e, f). In the postoperative MRI images, Achilles tendon got wider (arrowed site) at 12 months postoperatively (g, h). After percutaneous Achilles tendon lengthening, the right equinus when standing disappeared at 2 months postoperatively (i).

MRI results showed continuity of the Achilles tendon and homogeneous signal in group A. MRI showed fusiform-shaped tendon thickening postoperatively (Figure 5).

## 4. Discussion

The open Z-lengthening is an effective method for treatment of Achilles tendon contracture, but higher contracture recurrence rate and more complications can be frequently observed and dealt with difficulty [9]. Achilles tendon contracture is one part of the deformity in some complicate ankle deformity, and open Z-lengthening may increase the risk of incision complication. The patient needs to be placed at prone position when performing open Z-lengthening, while osteotomy and tendon transfer may be hindered at the same time. Percutaneous Achilles tendon lengthening has remarkable advantages of less trauma and reducing breakage of Achilles tendon blood supply, so it is globally accepted nowadays. But whether percutaneous Achilles tendon lengthening by triple hemisection can solve all the problems, especially in the primary operation, is still unknown from authoritative literatures. For the cases with low degree of Achilles tendon contracture, Achilles tendon lengthening is effective and safe. However, for the cases with severe Achilles tendon contracture, the risk of Achilles tendon rupture would be increased [5], and the full rupture may occur [12]. Complete Achilles tendon had not been indicated by some literatures of science and technology, but it was a severe complication [4, 9, 13–16]. The ankle and knee instability could occur if Achilles tendon was complete ruptured [17]. Because Achilles tendon lengthening could not be judged under direct vision, even the rupture position of Achilles tendon would not slide as a surgeon's wish. Hoefnagels et al. [18] reported irregular sliding of the fibers could also be observed in his study. Complete rupture of Achilles tendon in cross section is a serious complication. If not well solved, it would limit the popularization and application of this technique.

The method we promote in this study has two characteristics as a new percutaneous Achilles tendon lengthening. Firstly, it can better avoid the complication when Achilles tendon is completely transected. Achilles tendon is formed together from gastrocnemius and soleus tendon [19], and its extended area must be located on the two parts of tendon fiber area. When Achilles tendon lengthening by triple hemisection is adopted, if the distance between the two incisions was less than 5 cm, the risk of Achilles tendon fracture is increased for the serious case; if two hemi-cuts in the tendon was adopted (the site is near soleus) in our method and then sliding tendon, theoretically it can maximize the extension of the Achilles tendon. Secondly, Achilles tendon contracture is usually soft tissue imbalance which the ankle strength in plantarflexion is stronger than in dorsiflexion. It is helpful to avoid the recurrence when Achilles tendon strength in plantarflexion is weakened. So in this study, the most proximal incision was at the tendon-forming regions in gastrocnemius and the part below this region was the muscle tissue of soleus. Achilles tendon strength in plantarflexion can be weakened through cutting tension tendon tissue of gastrocnemius at most proximal incision. If this tension tendon is cut, it would have no obvious influence on the function of the ankle from our observation in clinical practice. Just as flatfoot associated with gastrocnemius contracture, cutting aponeurosis of gastrocnemius would have no influence on the function of the ankle. The fibers of the Achilles tendon has the phenomenon of rotation in three-dimensional space and there are significant individual differences in the rotation angles. The rotation can disperse the stress and avoid Achilles tendon injury; it may also increase the difficulty of percutaneous Achilles tendon lengthening. Achilles tendon torsion can lead to failure in Achilles tendon lengthening procedures [20]. Thus, the distance between the most distal incision and middle incision was controlled at 5 cm in our study. When the case is very serious (it is more than 5 cm), the surgeon should consider increasing the distance between two incisions. Because of the rotation, it's often difficult to pin down the proportion in the cross section and the degree of rotation, so for the safety of operation, the surgeon needs to cut more than half of tendon at first, then tries to do Achilles tendon lengthening. The width of the cuts is usually more than 50% of the tendon [6]. If the Achilles tendon is not lengthened, the cut range should be increased until the degree of ankle joint dorsiflexion increased over 100°. In a word, percutaneous Achilles tendon lengthening can be used in the primary operation, but the performer needs to have certain experience for serious Achilles tendon contracture. Patients with the recurrence of Achilles tendon lengthening didn't have a superb outcome; because of postoperative local tissue adhesion [21] and suture, the sliding tendon lengthening can be affected. For the revised cases, the open Z-lengthening is also recommended [22], and we do not recommend using percutaneous Achilles tendon lengthening in those patients.

Equinus recurrence rate needs to be evaluated in percutaneous Achilles tendon lengthening. In our study, only one patient suffered from equinus recurrence with percutaneous Achilles tendon lengthening and we believed that the reason of recurrence had no association with our operation itself. This patient suffered from equinus recurrence one year after surgery because of the infection at the fifth metatarsal, and then his feet were not on the floor for nearly 6 months. He did not do any function exercises during that time. So in our opinion, the function exercises are still very vital for these patients.

Previous studies on the clinical efficacy of Achilles tendon lengthening were mainly based on functional follow-ups and scores, and lacked direct imaging observation. MRI is a good tool in the healing process observation and postoperative rehabilitation [23, 24]. Achilles tendon shows hypointense signal on all imaging sequences [25]. When the Achilles tendon rupture occurres in the longitudinal direction, Achilles tendon images disappeares in transaxial planes. The T2 hyperintense signal may occur in uniform hypointense signal for Achilles tendon degeneration [26]. In the results, the gap could be observed in the longitudinal direction in sagittal planes on treatment of early postoperative period of Achilles tendon lengthening, while Achilles tendon images did not disappear in transaxial planes. That indicated that the Achilles tendon slided immediately after the operation while the tendon was not complete ruptured. As the rehabilitation training increased, tissue was found in the gap of lengthening gradually. Middle signal gradually became low signal in the MRI images, which indicated the fibrous tissue became tendon tissue. One year after operation, the MRI images showed uniform signal in Achilles tendon tissue and longitudinal continuity, suggesting a very good recovery, which means the Achilles tendon tissue was repaired completely. Interestingly, MRI images showed fusiform-shaped tendon thickening in some patients, indicating that the mechanism needed to be further studied. Some scholars [26, 27] also reported Achilles tendon volume increased at one year after treatment of Achilles tendon rupture.

No statistical difference was observed in therapeutic effect between the new percutaneous Achilles tendon lengthening and the traditional open Z-lengthening, but in new percutaneous Achilles tendon lengthening group, operative time and hospital stay were shorter and the incidence rate of incision complication was lower. The modified surgical method can significantly reduce the risk of Achilles tendon rupture; meanwhile, it has better balance in soft tissue strength between ankle dorsiflexion and ankle plantarflexion, helping to avoid recurrence of the deformity.

However, there are still some deficiencies in this study. Cases included are relatively few, and it is a retrospective analysis of a single center. These limitations would be solved in a multicenter randomized controlled trial in the future.

## Figures and Tables

**Table 1:** Characteristics of patients.

	Group A	Group B
Total pants (*n* = )	25	30
Sex		
Male	13	23
Female	12	7
Mean age (yr)	26.5 (6-57)	14.5 (5-43)
Side		
Right	6	14
Left	14	11
Bilateral sides	5	5

## Data Availability

The data used to support the findings of this study are available from the corresponding author upon request.
